# What is the best method for introducing complementary feeding?

**DOI:** 10.1590/1984-0462/2024/42/2023119

**Published:** 2023-10-03

**Authors:** Sabrine Teixeira Ferraz Grunewald

**Affiliations:** a Universidade Federal de Juiz de Fora, Juiz de Fora, MG, Brazil.

Dear editors:

The study by Moreira et al. elucidated the adherence to different strategies of introducing complementary feeding to infants, showing that, in the evaluated sample, baby-led introduction to solids (BLISS) had the lowest adherence — less than 20% of the families —, being often replaced by a mixed method.^1^ In this method, the caregiver is instructed to use the BLISS technique initially; however, if the child shows no interest in the food, the spoon is used to offer mashed food in the same meal.

The Brazilian Society of Pediatrics (*Sociedade Brasileira de Pediatria* — SBP) updated its guidelines on baby-guided food introduction methods in 2017, highlighting their growing popularity and dissemination on the internet and in books for parents. As noted by SBP experts, scientific research on these methods remains scarce, and studies are often conducted with a small sample population.^2^ Furthermore, we know that the food introduction process involves social, cultural, economic, and emotional issues, making the study by Moreira et al., conducted with Brazilian families, particularly welcome.

A Brazilian study with health professionals, mostly nutritionists, revealed that most of them recommend baby-led food introduction methods and believe in its potential advantages, although they emphasize discordant issues associated with the convenience of the method and allude to the concern/anxiety it can generate in caregivers.^3^

The outcomes of the study by Moreira et al. were evaluated at seven months of age, i.e., just one month after the recommended age for the introduction of food.^1^ Analyzing how the family behaves in association with the introduction methods as the child grows, develops, and takes on a new interest in different food shapes, textures, and flavors would be interesting. In addition, the families participating in the study were trained by health professionals in a workshop on the food introduction method that would be used, which unfortunately is not the reality in most Brazilian families.

Thus, as health professionals, we remain far from being able to recommend a specific method as optimal for introducing food to Brazilian children with promising evidence to support any such recommendation. Considering that each family has different needs, as healthcare professionals, it is important to stay updated with the evolving research on this subject.
