# Supplementary material for: What is the best method for introducing complementary feeding?
Source: Rev Paul Pediatr. 2023 Oct 3;42:e2023119. doi: 10.1590/1984-0462/2024/42/2023119 (PMC10593396; doi:10.1590/1984-0462/2024/42/2023119)
Supplement: Related texts

# Reply: What is the best method for introducing complementary feeding?

**Authors:** Paula Ruffoni Moreira, Leandro Meirelles Nunes, Renata Oliveira Neves, Christy Hannah Sanini Belin, Jordana Fuhr, Erissandra Gomes, Adriela Mariath, Juliana Rombaldi Bernardi

**Affiliations:** a Universidade Federal do Rio Grande do Sul, Porto Alegre, Rio Grande do Sul, Brazil.

Dear reader,

The authors appreciate your careful reading of our manuscript.^1^

The results published in this manuscript refer to the reported adherence by mothers at seven months of age of the child. Also, the authors agree that a longitudinal evaluation is necessary since the child's first year of life is marked by intense acquisition of skills that can significantly impact feeding. In the design of the randomized clinical trial, the authors planned to assess adherence to complementary feeding methods at seven, nine, and 12 months.^2,3^ However, the manuscript containing the complete data has not yet been published.

Furthermore, we emphasize that the mothers were unaware of the other methods and received the intervention they were randomized to. The planning of the randomized clinical trial occurred prior to the publication of the second edition of the *Feeding Guide for Brazilian Children Under Two Years Old*, which was published in 2019.^4^ The first edition^5^ did not include the recommendation for children to explore food with their hands, unlike the second, which does make this recommendation.

The authors agree that it is not common for families to receive training from healthcare professionals in a workshop to initiate food introduction methods with their children. This is both a limitation and a distinctive feature of our study.

Finally, the authors also agree that there is still insufficient evidence to support the recommendation of one complementary feeding method over another. Our research group is still analyzing and writing up the remaining results of the randomized clinical trial, and we hope to continue to contribute with high-quality scientific evidence to elucidate the topic.

The authors

## References

[1] Moreira PR, Nunes LM, Neves RO, Belin CHS, Fuhr J, Gomes E (2022). Adherence to different methods for introducing complementary food to 7-month-old babies: a randomized clinical trial. Rev Paul Pediatr.

[2] Nunes LM, Führ J, Belin CHS, Moreira PR, Neves RO, Brito ML (2021). Complementary feeding methods in the first year of life: a study protocol for a randomized clinical trial. Trials.

[3] Nunes LM, Führ J, Belin CHS, Moreira PR, Neves RO, Brito ML (2021). Correction to: complementary feeding methods in the first year of life: a study protocol for a randomized clinical trial. Trials.

[4] Brazil (2019). Guia alimentar para crianças brasileiras menores de 2 anos.

[5] Brazil (2013). Ministério da Saúde. Secretaria de Atenção à Saúde. Departamento de Atenção Básica. Dez passos para uma alimentação saudável: guia alimentar para crianças menores de dois anos: um guia para o professional da saúde na atenção básica.
